# Reverse-motion cervical rotation exercises improve cervical mobility: a 2-week intervention study

**DOI:** 10.3389/fspor.2026.1766547

**Published:** 2026-06-03

**Authors:** Koji Murofushi, Hiroshi Akuzawa, Sho Mitomo, Hiroki Katagiri, Takehiro Ohmi, Kazuyoshi Yagishita, Koji Kaneoka

**Affiliations:** 1 GENTEN Research Center, Institute of Science Tokyo, Tokyo, Japan; 2 Department of Orthopedic and Spinal Surgery, Graduate School of Medical and Dental Sciences, Institute of Science Tokyo, Tokyo, Japan; 3 Department of Rehabilitation, Sonoda Medical Institute Tokyo Spine Center, Tokyo, Japan; 4 Department of Orthopedics, Dokkyo Medical University Saitama Medical Center, Saitama, Japan; 5 Faculty of Sport Science, Waseda University, Tokyo, Japan

**Keywords:** activities of daily living, cervical range of motion, cervicothoracic mobility, corrective exercise, koji awareness, reverse motion, thoracic rotation

## Abstract

**Introduction:**

Cervical spine mobility is essential for daily functioning, sports performance, and overall spinal health. Among cervical spine motions, rotation is most strongly associated with limitations in activities of daily living (ADL). However, most corrective strategies rely on direct manipulation or stretching of the cervical spine. Reverse-motion exercises, which indirectly mobilize the cervical spine through thoracic and trunk movements, may provide a more effective and integrated alternative. This study examined the immediate and short-term effects of two reverse-motion cervical spine rotation exercises, Archer's Rotation and Python Squeeze, on cervical spine rotation range of motion (ROM).

**Methods:**

This study was a single-arm, 2-week intervention study. Participants first underwent the KOJI AWARENESS cervical spine mobility screening, and individuals who demonstrated rotational restrictions on this screening were selected as the study sample. A total of 17 participants (7 females, 10 males; mean age: 27.7 ± 5.8 years) completed the 2-week intervention. Cervical rotational ROM was assessed at baseline, immediately after the first exercise session, and after the 2-week intervention period. The program consisted of Archer's Rotation and Python Squeeze performed at home, with three sets of eight repetitions, 3 days per week.

**Results:**

Significant improvements were observed in both right and left cervical spine rotations. Right rotation increased from 60.6° ± 7.3° at baseline to 67.6° ± 7.1° immediately post-exercise and 68.5° ± 5.5° after 2 weeks (*p* < 0.001). Left rotation improved from 59.7° ± 5.4° to 66.5° ± 6.1° and 68.8° ± 6.0°, respectively (*p* < 0.001). The proportion of participants who achieved a full score on the KOJI AWARENESS cervical spine test also increased significantly after the intervention.

**Conclusion:**

The reverse-motion cervical spine exercises produced meaningful immediate and short-term improvements in cervical rotational ROM. These findings suggest that such exercises offer an effective, practical, and integrated method for enhancing cervical spine mobility.

## 1 Introduction

The cervical spine plays a crucial role as a musculoskeletal structure that supports the weight of the head, protects the upper spinal cord, houses the vertebral arteries supplying posterior circulation of the brain, and enables a wide range of head–neck movements (1, 2). Functionally, cervical spine motion is closely integrated with thoracic spine and rib cage mechanics (3) and is also linked to oculomotor control, because eye position and cervical spine muscle activity interact to regulate posture and coordinated movement (4).

Cervical spine-related problems have become increasingly prevalent in modern society. Lifestyle changes, particularly the prolonged use of smartphones and computers, impose sustained mechanical load on the cervical spine region and contribute to strain and postural dysfunction. Longer device use is a significant predictor of the duration of cervical spine and shoulder pain (5). Excessive smartphone use has been shown to alter sagittal and frontal spinal alignment (6), and forward-head posture associated with digital-device use is expected to increase cervical spine problems even among younger individuals (7). Muscle tension and poor cervical spine posture are also linked to headaches (8). In addition, cervical spine dysfunction is associated with autonomic dysregulation, anxiety, and depression, which further exacerbate neck pain (9). These clinical and psychosocial issues have been reported in both developed and developing countries and even among young populations (10, 11). Furthermore, cervical spine dysfunction has systemic implications, as individuals with chronic neck pain demonstrate reduced respiratory muscle strength and impaired pulmonary function (12).

Despite the increasing biomechanical and functional demand on the cervical spine, opportunities for intentional, functional cervical spine movements in daily life are declining. Among the main cervical spine motions—flexion, extension, lateral flexion, and rotation—limitations in rotation interfere most strongly with activities of daily living (ADL). Takeuchi et al. reported that rotational difficulties accounted for 41% of ADL limitations, compared with 34% for extension and 17% for flexion. Reduced cervical spine rotation range of motion (ROM) is also most strongly associated with postoperative ADL restriction (13). Collectively, these findings highlight cervical spine rotation as a central determinant of functional cervical spine mobility and independent daily living. Therefore, monitoring rotational function and improving cervical spine ROM are essential clinical objectives. Conventional interventions for cervical spine dysfunction include manual therapy (14, 15), active-release techniques (16), self-applied manual stretches, device-assisted traction-like movements (17), and direct voluntary neck movements (18). However, these approaches generally require direct manipulation or active movement of the head or cervical spine, which may be uncomfortable or difficult for some patients.

To address movement dysfunction more holistically, Murofushi et al. developed the KOJI AWARENESS system (KA), which consists of an 11-item movement screening test for motor function (19–21) and corresponding corrective exercises targeting specific dysfunctions (22–24). Within this system, two cervical spine exercises were devised using a reverse-motion-based method inspired by traditional Japanese performing arts, such as Noh and classical dance. These exercises differ fundamentally from conventional cervical spine interventions because they do not rely on direct head movement or manual stretching of the cervical region. Instead, this reverse-motion technique indirectly mobilizes the cervical spine through coordinated thoracic and scapular movements while keeping the head position. This approach may help inhibit excessive activation of the cervical spine rotatory muscles and facilitate more efficient cervicothoracic movement patterns. Although cervicothoracic coupling during axial rotation is well recognized, exercise interventions that restrict head movement while actively rotating the thoracic spine to induce relative cervical rotation have not been sufficiently investigated, particularly with respect to cervical rotation range of motion.

This preliminary study aimed to investigate how these indirect, non-head-directed exercises influence cervical spine ROM. We hypothesized that both Archer's Rotation and Python Squeeze would improve cervical rotation and may also promote cervical rotation ROM gains by enhancing thoracic–cervical spine interactions.

## 2 Materials and methods

### 2.1 Participants

Twenty-six participants were included in this study (11 females and 15 males; mean age: 29.5 ± 8.2 years; mean height: 169.1 ± 8.6 cm; mean weight: 72.2 ± 22.9 kg). Participants were recruited from a fitness center, and the study was conducted between October 2019 and March 2022.

The inclusion criteria were an age between 18 and 65 years and no medical conditions that would contraindicate exercise. The exclusion criteria were 1) a history of injury within the previous 3 months or 2) musculoskeletal or neurological problems that could impair exercise performance.

### 2.2 Study design and protocol

This study design was a single-arm, 2-week intervention study. KA self-screening of the cervical spine region was conducted for all participants, and individuals who demonstrated cervical spine mobility deficits based on the screening results were enrolled in the study group. After enrollment, baseline Cervical rotational ROM was measured.

Participants then received individualized instruction in corrective cervical spine exercises from an athletic trainer certified by the Board of Certification for the Athletic Trainer (ATC). Cervical rotational ROM was reassessed immediately after the initiation of corrective exercises.

Participants subsequently performed the corrective exercises at home for 2 weeks, after which cervical rotational ROM and KA self-screening scores the cervical spine components were measured again.

### 2.3 Procedures

The KA self-screening test consists of 11 components, including a cervical spine mobility assessment (20, 21). Each component requires the participant to perform a functional movement and assign a self-rating based on standardized criteria, yielding a total possible score of 50.

The cervical spine mobility assessment comprises four components with a maximum total score of six points, evaluating flexion, extension, lateral flexion, and rotation. Lateral flexion and rotation were assessed bilaterally.

Participants performed flexion, lateral flexion, and rotation in an upright standing posture with their hands placed on the waist.

**Figure 1:**
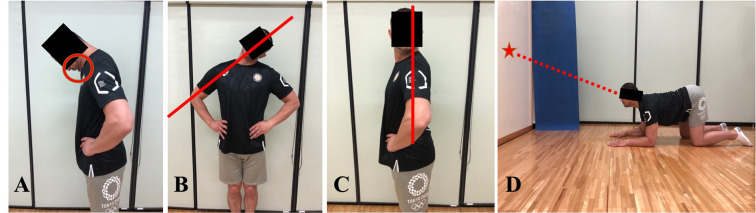
KA self-screening tests for the cervical spine part. **(A)** Flexion: flexing the cervical spine and bringing the chin toward the chest without opening the mouth. **(B)** Lateral flexion: laterally flexing the cervical spine while keeping the shoulders still, aligning the facial midline parallel to the upper arm. **(C)** Rotation: rotating the cervical spine while keeping the shoulders still, aligning the facial midline parallel to the shoulder line. **(D)** Extension: looking upward toward a marked point on the wall while maintaining the required posture.

- Flexion: Participants attempted to place their chins between their clavicles (Figure 1A). A score of 1 was assigned if this was achieved without opening the jaw.
- Lateral flexion: Participants attempted lateral cervical bending to the right and left (Figure 1B). A score of 1 for each direction was assigned if the facial midline remained parallel to the upper arm without cervical rotation.
- Rotation: Participants attempted to rotate the cervical spine to the right and left (Figure 1C). A score of 1 was assigned for each direction if the facial midline reached the center of the shoulder without lateral flexion or extension in the upright posture.
- Extension: Participants marked reference points on both the wall and the floor. The wall reference point was placed at a height equal to two lengths measured from the olecranon to the tip of the middle finger, taken from the floor. The floor reference point was placed at a distance of four steps away from the wall. Participants then positioned themselves so that their middle fingers rested on the floor mark and assumed a sphinx posture. From this position, they attempted to extend the cervical spine region to visually identify the mark on the wall (Figure 1D). A score of 1 was assigned if the participant could clearly see the wall mark while maintaining the proper posture.

Participants who scored less than 6 on the cervical spine components proceeded to the cervical rotational ROM assessment.

### 2.4 Cervical spine rotational range of motion test

Quantitative assessment of cervical rotational ROM was conducted to evaluate cervical spine mobility. Rotational ROM was prioritized because approximately 92% of rotational capacity is required for daily tasks such as backing up a car, whereas only about 30% of sagittal and frontal plane mobility is typically required (25).

Participants sat upright in a chair and maximally rotated the head to both sides while maintaining upright posture. An ATC measured active rotational ROM using a goniometer. Three measurements were taken per side, and the average value was used for analysis. Cervical rotation ROM measured with a goniometer demonstrates consistently high reliability, with Intraclass Correlation Coefficient (ICC2.2) ranges of 0.79–0.97 (26).

### 2.5 Self-exercises

#### 2.5.1 Archer's rotation

Archer's rotation is a dynamic cervical–thoracic spine mobility exercise inspired by the posture of drawing a bow. The exercise incorporates movement principles derived from traditional Japanese performing arts, such as Noh and classical dance, which emphasize a low center of gravity through slight knee flexion and activation of the *tanden* (lower abdominal center), promoting smoother upper-body motion.

**Figure 2:**
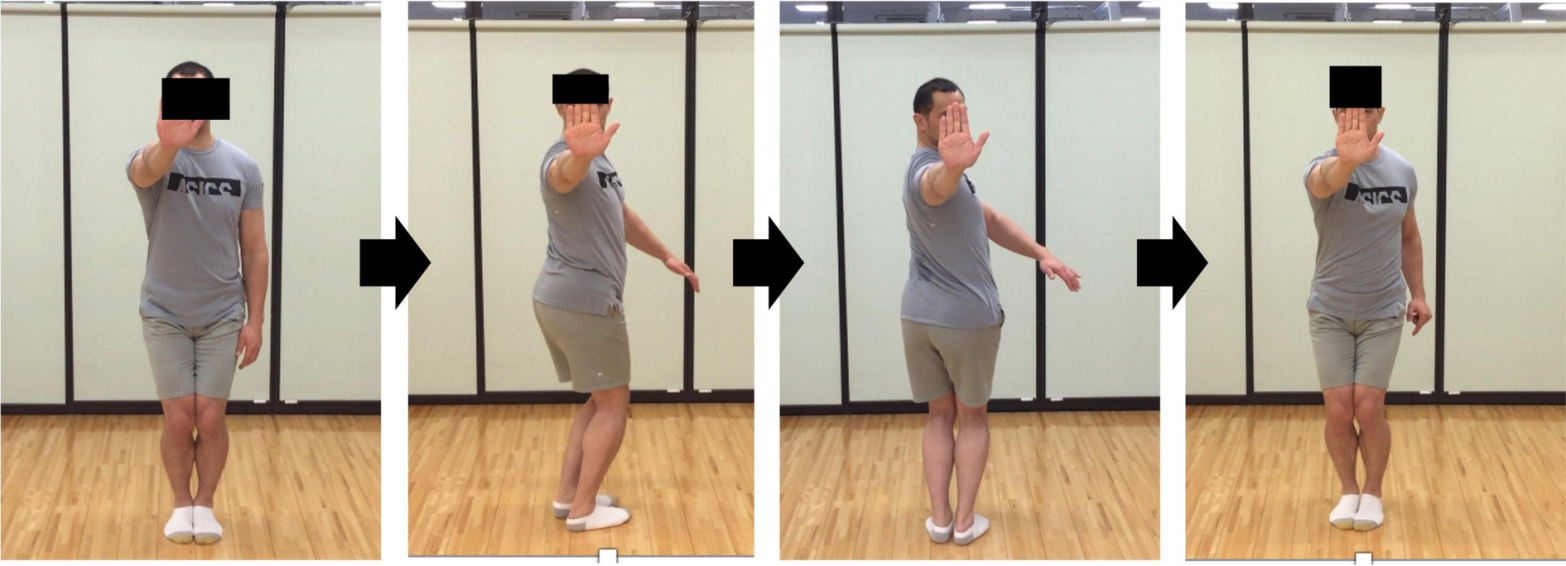
Archer's rotation. Instruction: bend the knees slightly while maintaining an upright upper body, and keep the gaze oriented toward the back of the elevated hand throughout the movement. Take small steps to one side until further rotation is no longer possible. Ensure that the alignment of the head and the back of the hand remain stable throughout the exercise.

Participants stood with knees slightly flexed and raised one arm to 90° shoulder flexion with the elbow fully extended. The wrist was dorsiflexed so that the dorsum of the hand aligned with the face. While maintaining head–hand alignment, participants performed rhythmic lateral steps toward the side opposite the elevated arm, allowing the trunk and thorax to rotate beneath the stable head–hand position (Figure 2). They then returned to the initial position without altering alignment. The exercise was performed in both directions.

#### 2.5.2 Python squeeze

**Figure 3:**
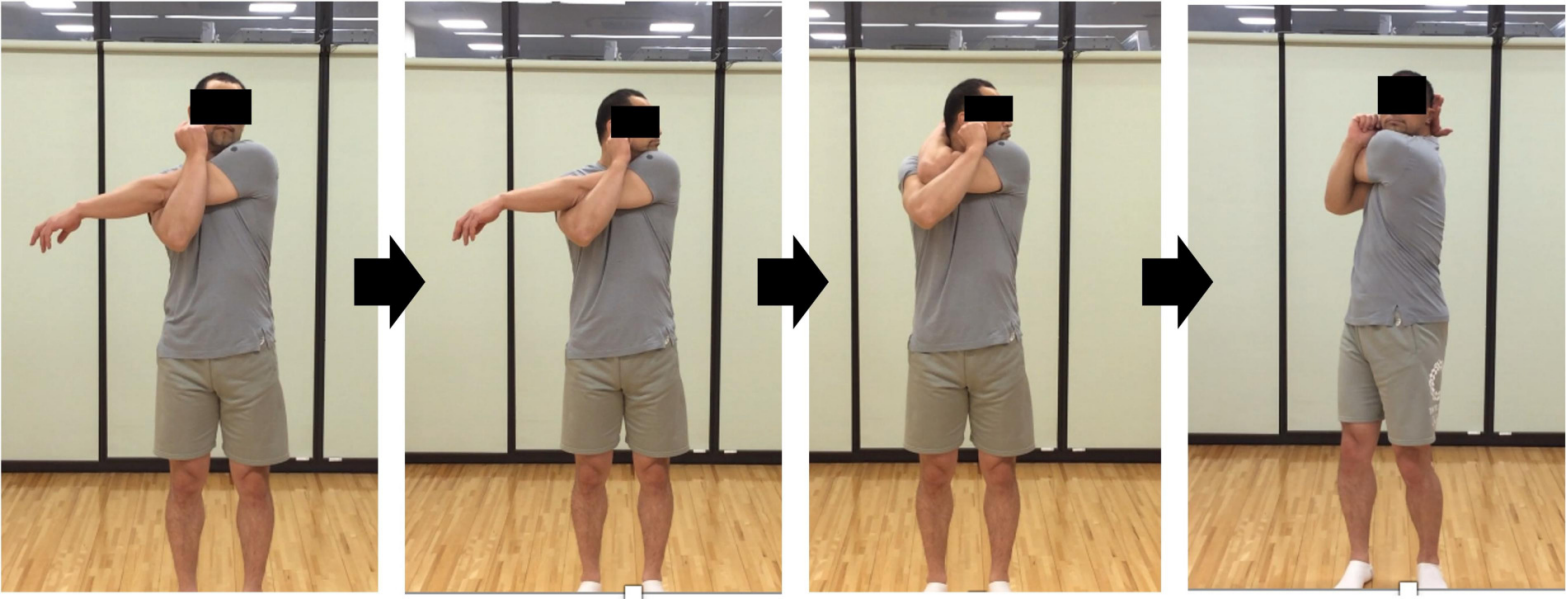
Python squeeze. Instruction: extend one arm straight, then use the opposite arm to bring it across the chest and curl it toward the body. Rotate the head rotate toward the direction of the extended arm. Bend the extended arm to reach behind the head with back of the hand (or reach the ear if flexibility allows). Rotate the entire upper body toward the side behind the head, hold for a few seconds, then unwind slowly and relax. Perform the movement slowly to feel the stretch and maintain proper breathing throughout the exercise.

Participants stood upright in a relaxed posture. One arm was brought across the chest into horizontal adduction, whereas the opposite arm was used to draw the adducted arm closer, creating a crossed-arm position. Participants then maximally rotated the cervical spine toward the side of the horizontally adducted arm. The assisting hand grasped the ear—or as close as possible—to stabilize the head (Figure 3).

Next, the elbow of the horizontally adducted arm was flexed so that the palm contacted the occipital area. Maintaining this alignment, participants rotated the trunk toward the side of the stabilized head until a stretching sensation was perceived, holding the position for several seconds. The return to neutral was performed in a controlled sequence: trunk, arms, then cervical rotation. Controlled breathing was encouraged to avoid abrupt relaxation. Each exercise was repeated eight times per set for a total of three sets.

#### 2.5.3 Monitoring adherence/compliance

To promote adherence to the exercise program, participants were provided with a checklist and instructed to record completion after each session. Correct execution of the exercises was confirmed through a form check conducted for all participants.

### 2.6 Statistical analyses

Statistical analyses were performed using SPSS Statistics, version 29 (IBM Corp., Armonk, NY, USA). The Shapiro–Wilk test assessed the normality of distributions. Depending on distribution characteristics, cervical spine ROM across time points was analyzed using either a one-way repeated-measures analysis of variance (for right rotation) or the Friedman test (for left rotation). When Mauchley's test indicated a violation of sphericity, the Greenhouse–Geisser correction was applied to adjust the F-statistic.

Post-hoc pairwise comparisons used Bonferroni adjustment. Effect sizes (ES) were calculated using partial eta-squared values. Changes in the proportion of participants achieving a perfect KA cervical spine region score (score of 6) were evaluated using Fisher's exact test. Statistical significance was set at *p* < 0.05.

Minimal detectable change (MDC) for the KA self-screening test for cervical spine mobility was calculated. Intra-rater reliability was assessed using the intraclass correlation coefficient [ICC (1, 3), two-way mixed-effects model, absolute agreement]. The standard error of measurement (SEM) was calculated as SD × √(1−ICC), and the minimal detectable change at the 95% confidence level (MDC95) was calculated as 1.96 × SEM × √2. These analyses were conducted using the individual item-level test–retest data provided in the Supplemental file of the previous study (27).

Because no external anchor was available, the minimal clinically important difference (MCID) was estimated using a distribution-based approach. The standard error of measurement was calculated using the baseline standard deviation and an intra-rater intraclass correlation coefficient derived from a previously published study employing the same cervical range-of-motion measurement procedure (26). To avoid side-selection bias, cervical rotation range of motion was summarized using the mean of left and right measurements.

### 2.7 Ethical approval

Ethical approval for this study was obtained from the Research Ethics Committee of our institution (M2019–168). All study procedures adhered to the principles outlined in the Declaration of Helsinki.

### 2.8 Informed consent

All participants were fully informed about the study's objectives and procedures and provided written informed consent prior to participation.

## 3 Results

### 3.1 Participants

**Figure 4:**
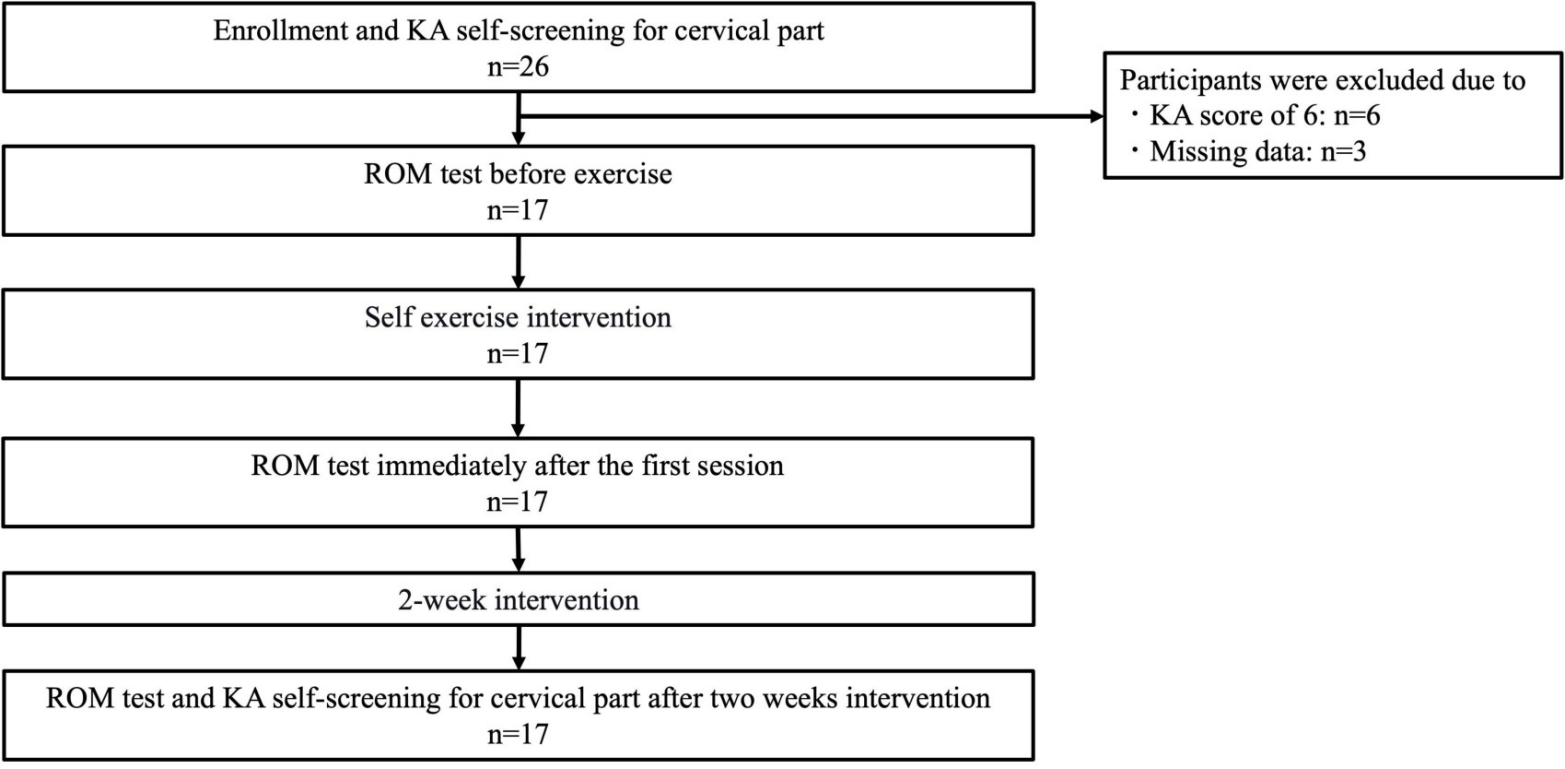
Flowchart of participant enrollment and changes in sample size throughout the study.

Six participants achieved a perfect score of 6 on the KA self-screening test for cervical spine mobility and were excluded from the study because they exhibited no cervical spine mobility restrictions. In addition, three participants were not able to participate in the cervical rotation ROM assessment due to scheduling conflicts. Thus, a total of 17 participants with cervical rotational motion restrictions were included in the study (7 females and 10 males; mean age: 27.7 ± 5.8 years; mean height: 170.9 ± 8.1 cm; mean weight: 73.5 ± 23.9 kg). All participants completed a 2-week exercise program, and their data were included in the statistical analysis (Figure 4). A *post-hoc* power analysis was performed using G*Power 3.1 for a repeated-measures ANOVA (within-subjects design). Using the observed effect size, an alpha level of 0.05, three repeated measurements, and a total sample size of 17, the estimated achieved power was high (1−*β* > 0.95).

### 3.2 Cervical rotation range of motion change

**Figure 5:**
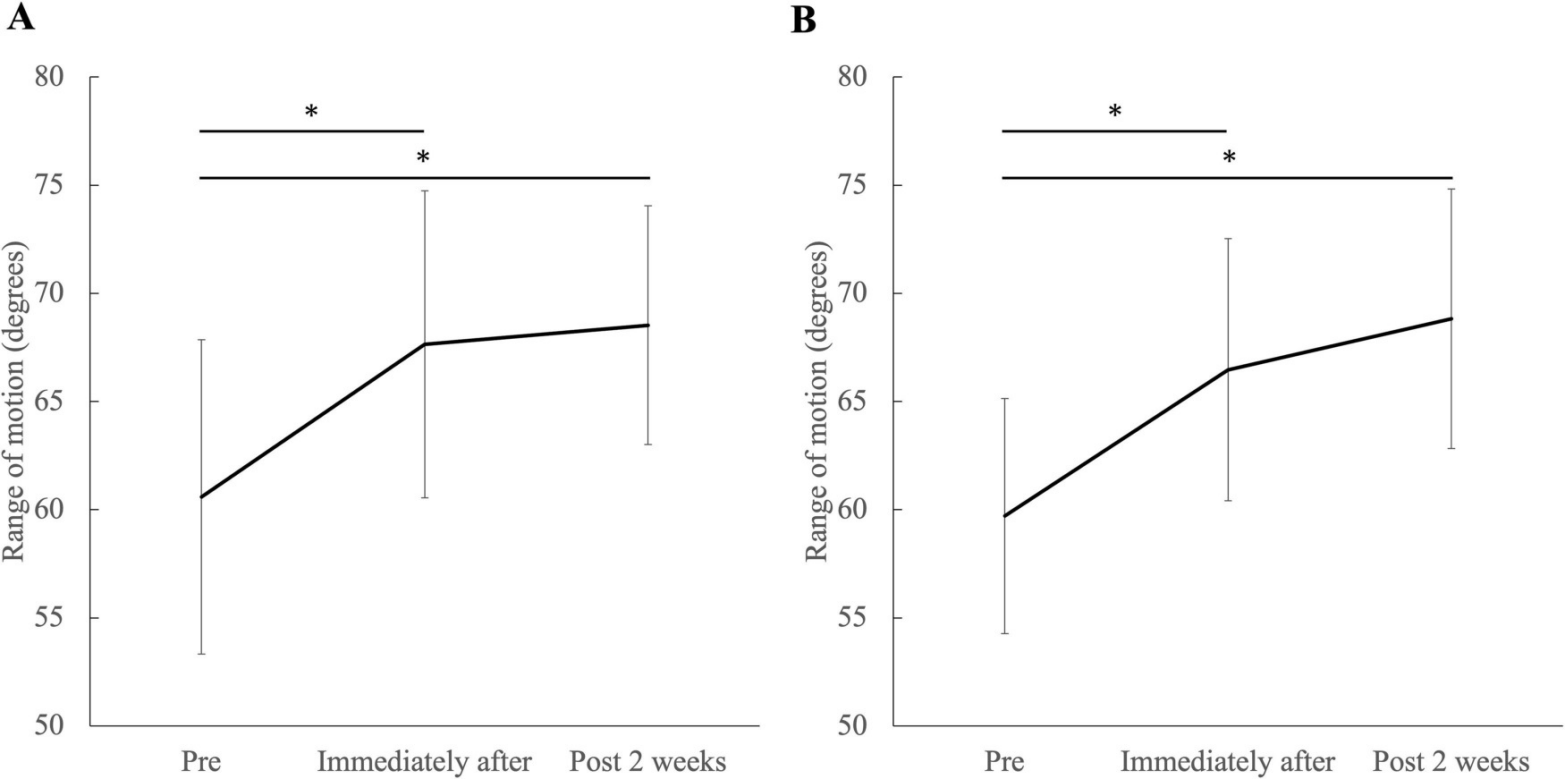
Changes in cervical rotational range of motion. **(A)** Right rotation. **(B)** Left rotation.

Changes in cervical rotation ROM are shown in Figure 5. The right and left rotational ROM values at each time point were as follows:

- Right rotation: pre-intervention: 60.6 ± 7.3°, immediately after the first exercise: 67.6 ± 7.1°, post-intervention (2 weeks): 68.5 ± 5.5°.
- Left rotation: pre-intervention: 59.7 ± 5.4°, immediately after the first exercise: 66.5 ± 6.1°, post-intervention (2 weeks): 68.8 ± 6.0°.

Significant main effects were observed for both right rotation (F_(2,32)_ = 10.08, *p* < 0.001, ES: 0.39) and left rotation (F_(2,32)_ = 19.61, *p* < 0.01, ES: 0.55). *post-hoc* analyses demonstrated that right and left rotational ROM significantly improved immediately after the first exercise session compared with pre-exercise values (*p* < 0.001, 95% confidence interval (95% CI): 1.2 to 12.9 for right rotation; *p* < 0.001, 95% CI: 2.8 to 10.7 for left rotation). Similarly, both right and left rotational ROM were significantly increased following the 2-week intervention (*p* < 0.001, 95% CI: 4.1 to 11.8 for right rotation; *p* < 0.001, 95% CI: 5.8 to 12.4 for left rotation). The corresponding MCID was estimated to be 5.7°.

### 3.3 KA self-screening mobility score

**Figure 6:**
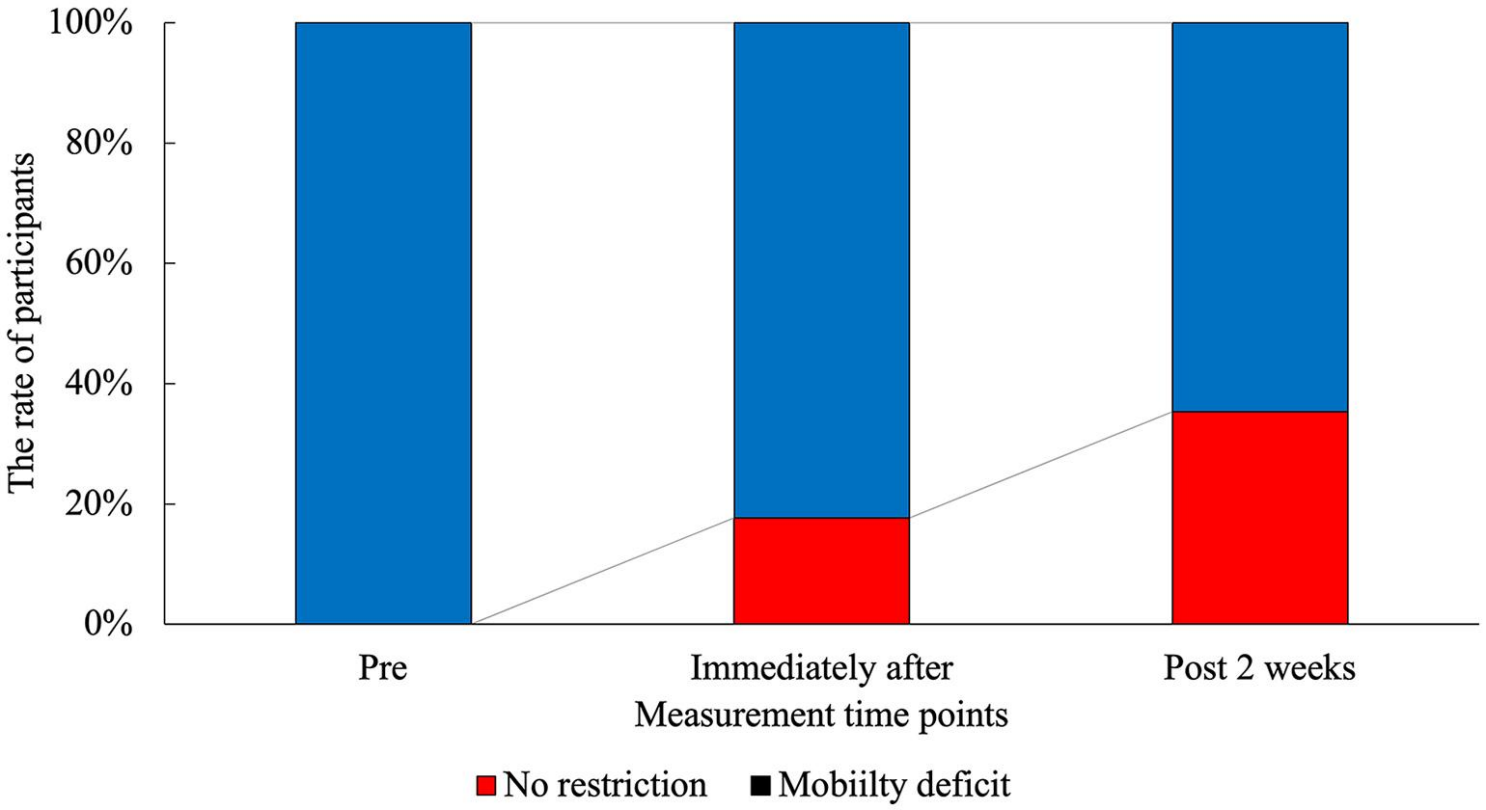
Proportion of participants who scored 6 on the KA self-screening cervical spine mobility test. Red indicates the rate of participants who scored 6; blue indicates the rate of participants who scored less than 5.

The ICC (1, 3) for the item was 0.63, indicating moderate reliability. The SEM was 0.83, and the MDC95 was 2.30. The proportion of participants who achieved a perfect score of 6 on the KA self-screening for the cervical spine region at each time point is shown in Figure 6. At pre-intervention, no participants obtained a score of 6 (mean score: 3.2 ± 1.3, range: 1–5). Three participants achieved a score of 6 immediately after the first exercise session, and six participants achieved a score of 6 after the 2-week intervention. Fisher's exact test revealed a significant association between the 2-week intervention and the proportion of participants achieving a perfect score (*p* = 0.03).

## 4 Discussion

The present study demonstrated that two cervical rotation-based exercises incorporating reverse motion, Archer's Rotation and Python Squeeze, produced both immediate and progressive improvements in cervical rotational ROM which exceed the estimated MCID. Participants with cervical rotation restrictions showed significant bilateral increases immediately after the first exercise session, with additional gains observed after the 2-week intervention period. The moderate effect sizes observed in both directions further support the clinical relevance of these changes. Furthermore, the increased proportion of participants who achieved a full score on the KA cervical spine mobility test indicates not only quantitative improvements in ROM but also qualitative enhancements in functional cervical spine mobility, such as smoother movement execution, reduced asymmetry between sides, and decreased reliance on compensatory motions.

Cervical rotation was selected as the primary intervention target because of its mechanical importance and substantial impact on daily function. Among cervical spine motions—flexion, extension, lateral flexion, and rotation—limitations in rotation most strongly interfere with ADLs. Takeuchi et al. reported that rotational difficulties accounted for 41% of ADL limitations, exceeding extension (34%) and flexion (17%) (15). Reduced cervical rotation ROM is also most strongly associated with postoperative functional restrictions. These findings highlight cervical spine rotation as a critical component of cervical spine mobility and justify prioritizing it in corrective exercise programs.

The immediate increase in cervical rotation range of motion observed after the first exercise session may be partially attributable to transient stretching or warm-up effects of the peri-cervical tissues. In contrast, the improvement observed after the 2-week intervention period may reflect early training-related adaptations, as the follow-up measurement was not performed immediately after the final self-exercise session. The mechanisms underlying the improvements observed in previous work using reverse-motion exercises—where thoracic rotation and scapular mobility were effectively enhanced (23, 24)—provide additional support for the present findings. Reverse-motion techniques promote joint mobility through indirect, thoracic-, or trunk-driven movements rather than through direct segmental activation. Extending this concept to the cervical spine region suggests that similar benefits can be achieved through cervicothoracic coupling, with thoracic motion contributing to improved cervical rotational capacity.

Traditional cervical interventions, including manual stretching, self-overpressure, and traction, primarily target the cervical spine region directly (16–18). In contrast, whereas Archer's Rotation and Python Squeeze indirectly promote cervical spine mobility by mobilizing the thorax, trunk, and lower body beneath a relatively stable cervical spine segment. The exercise strategy may reduce excessive reliance on superficial cervical rotators, such as the sternocleidomastoid and upper trapezius, while facilitating more coordinated activation of deep cervical stabilizers and thoracic rotators. Such a redistribution of movement demand could hypothetically enhance load sharing across the cervicothoracic junction, thereby contributing to more efficient rotational mechanics. Future studies incorporating biomechanical assessments, such as electromyography and three-dimensional motion analysis, are required to directly test these proposed mechanisms.

Archer's Rotation incorporates movement principles derived from traditional Japanese performing arts, such as Noh and classical dance, which emphasize lowering the center of gravity and cultivating awareness of the Tanden (lower abdominal center) (28). Archer's Rotation operationalizes these principles within a contemporary corrective exercise framework by maintaining a fixed head–hand alignment, while the lower body rotates beneath it. Lowering the hips minimizes unnecessary cervical spine contractions. Prior research on lower-body-driven trunk training demonstrated significant activation of deep stabilizers, including the multifidus and internal oblique muscles, contributing to enhanced trunk stability (29). Increased activation of these stabilizers may promote efficient load sharing throughout the cervicothoracic region, reducing excessive reliance on superficial cervical rotators during movement.

Python Squeeze complements these mechanisms by integrating thoracic rotation and rib cage mobility while keeping the head relatively stable. This movement pattern likely stretches the anterior chest and deep neck muscles while promoting activation of thoracic rotators, thereby improving segmental mobility in the thoracic spine—a region frequently implicated in cervical dysfunction (30). Given the close biomechanical interdependence of the cervical and thoracic regions, improvements in thoracic mobility may directly support gains in cervical rotation.

Together, these findings suggest that reverse-motion exercises emphasizing integrated cervical-thoracic–trunk coordination may provide a valuable alternative to conventional neck-focused interventions. These exercises enhance mobility without provoking excessive guarding and align with traditional Japanese movement principles emphasizing lower-body grounding, upper-body relaxation, and whole-body harmony.

Importantly, the cervical spine does not function independently; rather, its mobility is strongly influenced by thoracic mechanics and cervicothoracic coordination (31). Restriction of thoracic mobility is known to alter cervical mechanics, often resulting in compensatory muscle activation and reduced rotational capacity (32). The present findings highlight the potential benefit of shifting toward indirect, thoracic-driven strategies employing reverse motion to enhance cervical function.

The reverse-motion cervical spine exercises resulted in immediate and short-term improvements in cervical spine rotational ROM, which exceeded the estimated MCID, with a moderate effect size. The reverse-motion cervical exercises in this study produced meaningful immediate and short-term improvements in cervical rotational ROM. Because these tasks rely on thoracic- and trunk-driven movements rather than direct cervical rotation, they may be particularly suitable for individuals who experience pain, stiffness, or apprehension during head rotation. The exercises are simple, require no equipment, and can be performed safely at home, making them practical and accessible options for clinicians seeking effective approaches to improve cervical mobility and functional capacity.

This study has several limitations. First, this study did not include a control group, and no comparison was made with commonly used cervical spine mobility exercises; therefore, it remains unclear whether the reverse-motion exercises are superior or equivalent to conventional approaches. Second, the study did not examine potential learning effects associated with repeated assessments, making it impossible to rule out the possibility that part of the observed improvements in ROM were influenced by familiarity with the testing procedure. Third, all assessments were performed by a single evaluator without blinding, which may have introduced measurement bias. Fourth, because participant inclusion was determined based on the Koji awareness screening, there is a possibility of selection bias, which should be considered when interpreting the results. Finally, the study did not assess changes in ADLs, pain, function such as strength, leaving unanswered whether improvements in ROM translate into functional benefits. Future research should incorporate comparison groups, symptom-related measures, and functional outcomes to clarify the broader clinical utility of these exercises.

In conclusion, the present study demonstrated that two reverse-motion exercises, Archer's Rotation and Python Squeeze, can produce meaningful immediate and 2-week improvements in cervical rotational ROM. These exercises enhance mobility through cervicothoracic integration rather than direct cervical spine movement, suggesting an accessible and potentially effective method for improving cervical spine function. Future studies should compare these interventions with conventional cervical spine exercises and investigate long-term clinical outcomes.

## Data Availability

The datasets generated and/or analyzed during the current study are available from the corresponding author on reasonable request and in accordance with institutional and ethical guidelines.

## References

[1] Bogduk N. Functional anatomy of the spine. Handb Clin Neurol. (2016) 136:675–88. 10.1016/b978-0-444-53486-6.00032-6 27430435

[2] Rahman S Das JM. Anatomy, head and neck: cervical spine. In: StatPearls. Treasure Island, FL: StatPearls Publishing (2025). Available at: Available online at: https://www.ncbi.nlm.nih.gov/books/NBK557516/ (Accessed August 28, 2023). 32491448

[3] Endo K Suzuki H Sawaji Y Nishimura H Yorifuji M Murata K. Relationship among cervical, thoracic, and lumbopelvic sagittal alignment in healthy adults. J Orthop Surg (Hong Kong). (2016) 24:92–6. 10.1177/230949901602400121 27122521

[4] Bexander CS Mellor R Hodges PW. Effect of gaze direction on neck muscle activity during cervical rotation. Exp Brain Res. (2005) 167:422–32. 10.1007/s00221-005-0048-4 16193272

[5] Maayah MF Nawasreh ZH Gaowgzeh R Neamatallah Z Alfawaz SS Alabasi UM. Neck pain associated with smartphone usage among university students. PLoS One. (2023) 18:e0285451. 10.1371/journal.pone.0285451 37352232 PMC10289365

[6] Betsch M Kalbhen K Michalik R Schenker H Gatz M Quack V. The influence of smartphone use on spinal posture – a laboratory study. Gait Posture. (2021) 85:298–303. 10.1016/j.gaitpost.2021.02.018 33640863

[7] Öğrenci A Koban O Yaman O Dalbayrak S Yılmaz M. The effect of technological devices on cervical lordosis. Open Access Maced J Med Sci. (2018) 6:467–71. 10.3889/oamjms.2018.107 29610602 PMC5874367

[8] Matharu M Katsarava Z Buse DC Sommer K Reed ML Fanning KM. Characterizing neck pain during headache among people with migraine: multicountry results from the CaMEO-I study. Headache. (2024) 64:750–63. 10.1111/head.14753 38982663

[9] Alghamdi MS Alghamdi AF Almalawi AM Alsulami RA Hazazi HA Al Ghashmari AA. The association between neck pain and psychological distress among university students: a cross-sectional study. Cureus. (2023) 15:e35685. 10.7759/cureus.35685 37012948 PMC10066660

[10] Demyttenaere K Bruffaerts R Lee S Posada-Villa J Kovess V Angermeyer MC. Mental disorders among persons with chronic back or neck pain: results from the world mental health surveys. Pain. (2007) 129:332–42. 10.1016/j.pain.2007.01.022 17350169

[11] Dighriri YH Akkur MA Alharbi SA Madkhali NA Matabi KI Mahfouz MS. Prevalence and associated factors of neck, shoulder, and low-back pain among medical students. J Family Med Prim Care. (2019) 8:3826–31. 10.4103/jfmpc.jfmpc_721_19 31879620 PMC6924257

[12] López-De-Uralde-Villanueva I Del Corral T Salvador-Sánchez R Angulo-Díaz-Parreño S López-Marcos JJ Plaza-Manzano G. Respiratory dysfunction in patients with chronic neck pain: systematic review and meta-analysis. Disabil Rehabil. (2023) 45:2422–33. 10.1080/09638288.2022.2096126 35802487

[13] Takeuchi K Yokoyama T Ono A Numasawa T Wada K Kumagai G. Limitations of ADL accompanying reduced neck mobility after cervical laminoplasty. Arch Orthop Trauma Surg. (2007) 127:475–80. 10.1007/s00402-007-0372-1 17581759

[14] Ceylan İ Canlı M Kuzu Ş Ayhan DT Gürses ÖA Oyman BE. The effectiveness of two different treatment approaches in individuals with chronic non-specific neck pain: a randomized control trial. Turk J Health S. (2023) 4(2):56–62. 10.29228/tjhealthsport.69835

[15] Kocaman H Yıldız NT Canlı M Alkan H. Comparison of the effects of mulligan mobilization technique combined with cervical stabilization exercises with the effects of cervical stabilization exercises alone in chronic neck pain: randomized controlled study. Karya J Health Sci. (2023) 4(3):227–34. 10.52831/kjhs.1374767

[16] Cho J Lee E Lee S. Upper thoracic spine mobilization versus upper cervical mobilization in forward head posture. BMC Musculoskelet Disord. (2017) 18:525. 10.1186/s12891-017-1889-2 29233164 PMC5727966

[17] Sun X Chai L Huang Q Zhou H Liu H. Effects of exercise combined with cervicothoracic self-mobilization on chronic non-specific neck pain. Sci Rep. (2024) 14:5298. 10.1038/s41598-024-55181-8 38438448 PMC10912754

[18] Lee J Lee M Lim T Kim T Kim S Suh D. Effectiveness of an app-based neck exercise for chronic neck pain: a pilot randomized trial. Eur J Integr Med. (2017) 12:87–92. 10.1016/j.eujim.2017.04.012

[19] Murofushi K Katagiri H Mitomo S Hirohata K Furuya H Hanazawa R. Exploring age-related changes in motor function using the koji awareness screening test. Sci Rep. (2024a) 14:18903. 10.1038/s41598-024-69971-7 39143124 PMC11324887

[20] Murofushi K Yamaguchi D Katagiri H Hirohata K Furuya H Mitomo S. Validity of the koji awareness self-screening test for body movement. PLoS One. (2022c) 17:e0277167. 10.1371/journal.pone.0277167 36584031 PMC9803145

[21] Murofushi K Yamaguchi D Katagiri H Hirohata K Furuya H Mitomo S. Relationship between movement self-screening scores and pain intensity during training. J Med Invest. (2022b) 69:204–16. 10.2152/jmi.69.204 36244771

[22] Murofushi K Yamaguchi D Kaneoka K Oshikawa T Katagiri H Hirohata K. Effectiveness of corrective exercises on KOJI AWARENESS score and activity-related pain. J Med Invest. (2023) 70:208–12. 10.2152/jmi.70.208 37164722

[23] Murofushi K Mitomo S Hirohata K Furuya H Akuzawa H Katagiri H. Sequential changes in scapular ROM after the KOJI AWARENESS “wall Angel slider” exercise. J Sport Rehabil. (2025) 35(1):1–7. 10.1123/jsr.2024-0426 40348392

[24] Murofushi K Mitomo S Hirohata K Furuya H Katagiri H Kaneoka K. Comparative analysis of thoracic rotation exercises: standing vs quadruped. Acta Med Okayama. (2024b) 78:251–8. 38902213 10.18926/AMO/67200

[25] Bible JE Biswas D Miller CP Whang PG Grauer JN. Normal cervical ROM during 15 ADL. J Spinal Disord Tech. (2010) 23:15–21. 10.1097/BSD.0b013e3181981632 20051924

[26] Farooq MN Mohseni Bandpei MA Ali M Khan GA. Reliability of the universal goniometer for assessing active cervical range of motion in asymptomatic healthy persons. Pak J Med Sci. (2016) 32(2):457–61. 10.12669/pjms.322.8747 27182261 PMC4859044

[27] Takasaki H Kanayasu S. Koji awareness, a self-rating whole-body movement assessment system, has intersession reliability and comparability to external examiner rating. PLoS One. (2024) 19(8):e0308973. 10.1371/journal.pone.0308973 39146306 PMC11326541

[28] Habu H Mitsuhashi T Tokinobu A Yorifuji T Takao S. Effects of tanden breathing on constipation: a randomized trial. Acta Med Okayama. (2022) 76:391–8. 36123153 10.18926/AMO/63893

[29] Murofushi K Oshikawa T Kaneoka K Akuzawa H Yamaguchi D Mitomo S. Differences in trunk and leg muscle activity during squatting with and without hammer swing. Sci Rep. (2022a) 12:13387. 10.1038/s41598-022-17653-7 35927570 PMC9352780

[30] Lau KT Cheung KY Chan KB Chan MH Lo KY Chiu TT. Relationships between thoracic/cervical sagittal posture and neck pain. Man Ther. (2010) 15:457–62. 10.1016/j.math.2010.03.009 20430685

[31] Tsang SM Szeto GP Lee RY. Normal kinematics of the neck: interplay between cervical and thoracic spine. Man Ther. (2013) 18:431–7. 10.1016/j.math.2013.03.002 23632368

[32] Seo J Song C Shin D. Thoracic manipulation vs mobility exercises in office workers with chronic neck pain: a randomized trial. Med Sci Monit. (2022) 28:e937316. 10.12659/msm.937316 35799408 PMC9275077

